# Comprehensive Analysis of Bioactive Compounds in Wild *Ganoderma applanatum* Mushroom from Kerala, South India: Insights into Dietary Nutritional, Mineral, Antimicrobial, and Antioxidant Activities

**DOI:** 10.3390/ph17040509

**Published:** 2024-04-17

**Authors:** Akbar Rijia, Raman Krishnamoorthi, Madhusoodhanan Rasmi, Pambayan Ulagan Mahalingam, Kwang-sun Kim

**Affiliations:** 1 Department of Biology, The Gandhigram Rural Institute (Deemed to be University), Gandhigram, Dindigul 624302, Tamil Nadu, India; rijiazareen@gmail.com; 2 Pharmaceutics Laboratory, Graduate Institute of Natural Products, Chang Gung University, Kweishan, Taoyuan 33302, Taiwan; krishmicro3@gmail.com; 3 Department of Microbiology, Bharathidasan University, Tiruchirappalli 620024, Tamil Nadu, India; rasmi.m@bdu.ac.in; 4 Department of Chemistry and Chemistry Institute for Functional Materials, Pusan National University, Busan 46241, Republic of Korea

**Keywords:** *Ganoderma applanatum*, antioxidant activity, bioactive compound, antimicrobials

## Abstract

The present study focused on the mushroom Ganoderma, which has been used in Eastern countries for centuries as a food and medicinal source. Specifically, the fruiting bodies of *Ganoderma applanatum* from the Kerala Forest Research Institute in Thirussur, Kerala, India, were analyzed for their nutritional and medicinal properties. The methanolic extracts of *G. applanatum* were used to examine secondary metabolites and proximate profiles, revealing the presence of various phytochemicals such as terpenoids, phenolics, glycosides, alkaloids, flavonoids, and saponins. Further analysis revealed the presence of significant amounts of calcium, sodium, phosphorus, and manganese. The compounds were characterized using chromatographic analysis, FTIR, and GC-MS, which revealed potential therapeutic compounds with C-H and C-O bonds in the amide group, β-glycosides, and C-C/C-O vibrations of phenolic substances. Mushroom extract at a concentration of 100 µg mL^−1^ exhibited potent antimicrobial activity against various pathogens. This study suggests that *G. applanatum* has a rich biochemical composition and pharmacological potential, making it a promising candidate for drug development and traditional medicine, and contributes valuable insights into its diverse therapeutic applications.

## 1. Introduction

Mushrooms are a diverse group of macrofungi that are a significant component of forest ecosystems and occupy a prominent position in the biological world because of their clinical, agricultural, food, environmental, and economic importance. The dual significance of mushrooms as both edible delights and potent medicinal agents has spurred growing interest among researchers and health enthusiasts alike [1]. Culinary mushrooms are excellent sources of nutrition because they contain secondary metabolites, polysaccharides, minerals, unsaturated fatty acids, and proteins. Vitamins, such as riboflavin, thiamin, folic acid, ascorbic acid, and ergosterol, and minerals, such as phosphorus, selenium, magnesium, and copper, are widely distributed throughout the fruiting bodies of many mushrooms. Fruiting bodies are abundant in β-glucans, chitin, and dietary fibers [2,3,4,5].

Both wild and farmed mushrooms are now staples of the human dietary regimen. To date, there are several wild mushrooms that can be considered edible, and most of them are consumed worldwide owing to their desirable taste, flavor, texture, specific aroma, and a wide range of organic compounds with therapeutic and dietary benefits [6,7]. Several investigations have presented different mushrooms as an abundant supply of biologically active substances, which could be helpful in reducing the risk of illnesses, such as several types of cancer, stroke, Parkinson’s disease, Alzheimer’s disease, and hypertension [8,9,10]. Certain types of mushrooms contain fruiting body extracts that have antimicrobial, immune-boosting, and cholesterol-reducing effects [2,4,11]. Many polysaccharides from a variety of mushroom species belonging to the Agaricaceae, Polyporaceae, and Tricholomataceae families have remarkable in vivo antitumor activity [12,13,14]. Over the last few decades, Oriental and ancient therapies have been extensively used to track the records of therapeutic mushrooms. Combinations of several therapeutic mushrooms, such as *Auricularia*, *Flammulina*, *Ganoderma*, *Grifola*, *Hericium*, *Lentinus*, *Pleurotus*, *Schizophyllum*, *Trametes*, and *Tremella* are used for maximum benefit [15,16]. Edible mushrooms *Flammulina velutipes*, *Grifola frondosa*, *Lyophyllum shimeji*, and *Pleurotus eryngii* have all been shown to possess health-promoting and therapeutically active substances [17]. However, some effective, palatable therapeutic mushroom types, such as *Ganoderma lucidum*, *Inonotus obliquus*, and *Trametes versicolor*, are extremely sour or challenging to consume and are thus utilized as extracts, syrups, drinks, spore products, dietary supplements, coffee, and tea powder [18,19]. Additionally, a range of goods including lotions, soaps, and toothpastes are made from the fruiting bodies of *Ganoderma* and marketed as useful dietary and pharmaceutical additives with health advantages [20].

*Ganoderma* spp. are among the most widely studied medicinal fungi. Poroid wood-degrading macrofungi belonging to the genus *Ganoderma* are mostly found in tropical and subtropical forests worldwide. These mushrooms often grow as saprophytes or parasites on live trees. However, they can occasionally be observed attached to tree stumps and woodlogs. The genus *Ganoderma* has a very complex taxonomy. There are approximately 80 *Ganoderma* species worldwide, and approximately 117 species have been recorded in the family *Ganodermataceae* of Basidiomycota [20]. Certain *Ganoderma* species can break down cellulose, lignin, and various other associated polysaccharides in both angiosperms and gymnosperm hosts, leading to various rot diseases. Many woody trees of different *Ganoderma* species are declining because of stem and root rot. This is one of the advantages of identifying the *Ganoderma* spp. *Ganoderma* spp. have a diverse variety of phytochemicals, including alkaloids, amino acids, fatty acids, phenolic compounds, proteins and polysaccharides, steroids, and terpenoids, with advantages in food and medicine, including antifungal, antibacterial, antiviral, antidiabetic, antioxidant, immunomodulatory, anti-inflammatory, anti-adipogenic [21], and anticancer properties [22,23,24,25,26]. This has led to a great deal of curiosity regarding in-depth analysis of this mushroom. Therefore, it should be promoted as an effective food supplement for maintaining health.

Wild medicinal mushrooms have been extensively studied by many ethnobotanists and medical researchers in other parts of the world, whereas southern India has been relatively less explored and unexploited. The Western Ghats, one of the hotspots of biological diversity, runs parallel to the west coast of India from Maharashtra, South Gujarat, Goa, Karnataka, Kerala, and Tamil Nadu, covering up to 160,000 km^2^ [27]. However, different types of farmed mushrooms are well known in this region because of their edibility, nutritional importance, and therapeutic effects. Numerous edible mushroom species that bloom in the forest have to be eaten by various tribes in the southern Indian region because of their exotic flavors and tastes, without knowing their nutritional and medicinal importance [28,29,30]. In India, several cases of misidentification of fruiting bodies collected from the wild for consumption were reported in different states between 2010 and 2019 [31,32]. However, the dietary significance, toxicity, and medicinal properties of mushrooms need to be explored. In addition, the identification of the most promising species of mushrooms with high nutritive value, development of agrotechnology, and extension of its cultivation technologies among rural growers, unemployed youth, women-folk, and weaker populations may increase the economic status of people in the southern Indian region.

The number of well-researched mushrooms needs to be improved, even among the recognized species. However, further research is needed to capitalize on these beneficial medicinal properties. Genus *Ganoderma* is a well-explored bioactive secondary-metabolite-rich mushroom with medicinal effects. Extensive research has been conducted on the laccate species (*G. lucidum*). However, very few efforts have been made to explore the non-laccate species (*G. applanatum*) found in Kerala, South India. Pharmacognostic standards must be set before any crude medicine can be successfully bioprospected into herbal pharmacopeia [33]. Despite this, attempts have been made to study the nutritional, chemical, and therapeutic properties of *G. applanatum* in Kerala, South India.

## 2. Results

### 2.1. Collection of Wild Mushroom G. applanatum

**Figure 1:**
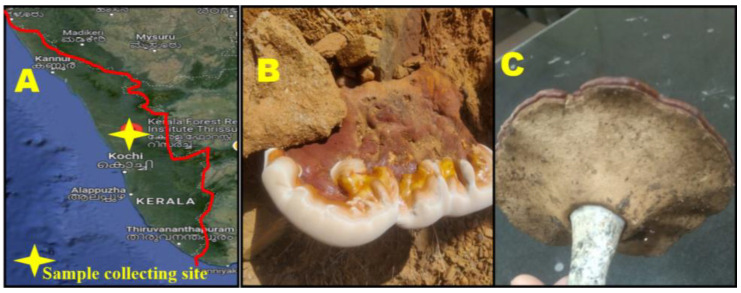
Collection of *G. applanatum*. (**A**) Collection site (source: Google map of India), (**B**) fruiting bodies outer side, and (**C**) fruiting bodies inner side.

In the present study, 31 fruiting body samples were obtained from different locations in the Kerala Forest Research Institute (KFRI), Peechi, Thrissur, and Kerala (Figure 1A–C). Throughout this investigation, most of the mushroom samples were collected from the soil, followed by decaying wood and other natural sources. 

### 2.2. Proximate Composition of G. applanatum Mushroom

**Figure 2:**
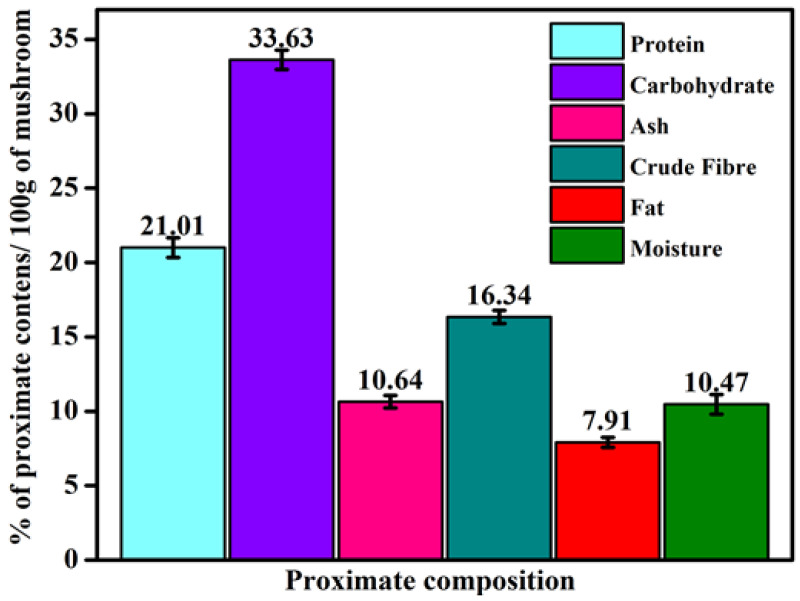
Proximate profile revealing the nutrient constitution of *G. applanatum* mushroom extracts. Values are the mean ± SD from *n* = 3.

In this study, the proximate composition of the collected mushrooms considered for evaluation was moisture, ash, fiber, crude protein, carbohydrate, and fat content. The results of this study showed that the *G. applanatum* mushroom has superabundant nutrients, such as carbohydrates (33.63 ± 0.66%) and proteins (21.01 ± 0.88%) (Figure 2). The total energy value was 262.50 ± 2.12 calories per 100 g of this mushroom. Mushrooms possess a rich amount of crude fibers (16.34 ± 0.33%) and insoluble and soluble (β-glucans and chitosan) fibers. Mushrooms have a low amount of beneficial n-3 fatty acids and a minimal total lipid level (7.91 ± 0.33%). Therefore, they have a limited nutritional contribution and are better for human diets and cardiovascular conditions. In our study, the ash and moisture contents were 10.64 ± 0.66% and 10.47 ± 1.12%, respectively. These findings suggest that *G. applanatum* is an excellent source of ash, carbohydrates, fiber, and protein. 

### 2.3. Qualitative Analysis of Phytochemicals from G. applanatum

The current findings concern the presence of secondary metabolites in *G. applanatum* mushrooms in the Western Ghats of Kerala. Qualitative phytochemical screening of the crude methanol extracts of *G. applanatum* revealed the presence of alkaloids, flavonoids, phenolic substances, saponins, and terpenoids (Table 1).

### 2.4. Quantitative Analysis of Phytochemicals from G. applanatum

The quantification of phytochemicals revealed that *G. applanatum* mushrooms possess significant amounts of phenolic substances [22.9 mg of gallic acid equivalent (GAE)] and flavonoid substances [15.84 mg of quercetin equivalent (QE)] per gram in the mushroom sample (dry weight). Moreover, terpenoid substances and saponin were present in 0.351 mg and 22.19 µg per gram and milligram, respectively, in dry *G. applanatum* mushrooms (Table 2).

### 2.5. Minerals Constitution in G. applanatum Mushroom

Atomic absorption spectrometry (AAS) was performed using the ash remaining after the whole burning of the dried *G. applanatum* mushroom and found the elemental minerals of the fruiting bodies. As shown in Table 3, calcium (Ca; 283.00 ± 3.12 mg kg^−1^), sodium (Na; 190.00 ± 2.76 mg kg^−1^), phosphorus (P; 117.00 ± 1.12 mg kg^−1^), and a moderate level of potassium (K; 23.9 ± 0.66 mg kg^−1^) were present in *G. applanatum* mushroom. Additional AAS analysis of four micronutrients including zinc (Zn), copper (Cu), manganese (Mg), and iron (Fe) showed a remarkable elevation of Zn (10.34 ± 1.27 mg kg^−1^), whereas the other two elements [Fe (4.83 ± 0.33 mg kg^−1^) and Mg (4.6 ± 0.66 mg kg^−1^)] were present in a low amount, which was in accordance with the results of previous studies [34,35]. A negligible amount of Cu (0.781 ± 0.88 mg kg^−1^) was also detected.

### 2.6. Elucidation and Characterization of Compounds from G. applanatum Mushroom Extracts

#### 2.6.1. Thin-Layer Chromatography (TLC) Profile

**Figure 3:**
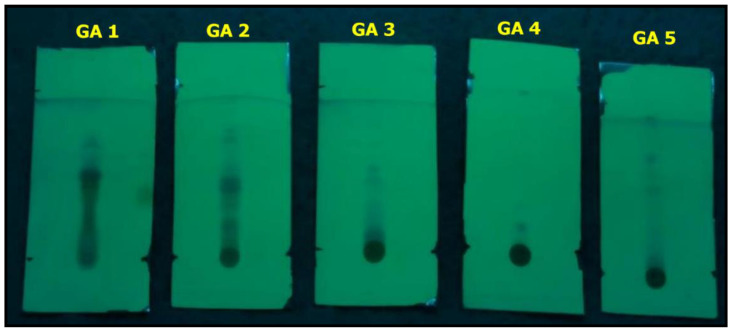
TLC profile of various bioactive compounds in *G. applanatum* mushroom extract.

**Figure 4:**
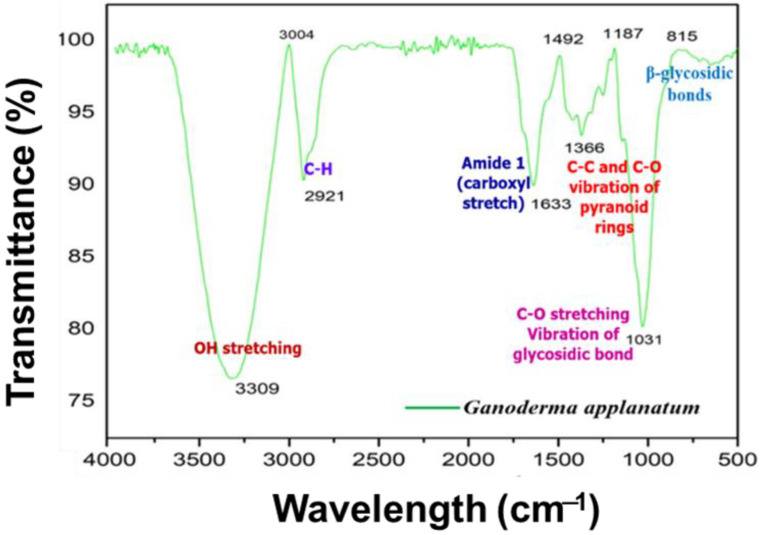
Functional group analysis of *G. applanatum* mushroom extract via FTIR spectroscopy.

TLC analysis suggested the presence of alkaloid fractions, flavonoids, glycosides, phenols, and terpenoids (R_f_ values: 0.720, 0.155, 0.560, 0.809, and 0.534, respectively) in the *G. applanatum* mushroom (Figure 3; Table 4). This finding was further supported by qualitative and quantitative Fourier-transform infrared (FTIR) spectroscopy and gas chromatography–mass spectrometry (GC-MS) analyses (Figure 4; Table 5). Remarkably, the corresponding alkaloid, flavonoid, terpenoid, glycoside, and phenol fractions were green, blue, blue, blue, and blue in color, respectively; therefore, the specific conforming band was not noticeable in the TLC plates.

#### 2.6.2. FTIR Analysis

Figure 4 shows the FTIR spectrum of the methanol extract of the *G. applanatum* mushrooms. The large absorption peak was the OH stretching peak and C-H was 3309.00 cm^−1^ and 2921.00 cm^−1^, respectively. The absorption bands at 1633.00 cm^−1^ and 1366 cm^−1^ were attributed to the stretching vibration of the carboxyl bond (C-O) of the amide group and the C-C and C-O vibrations of the phenolic substance or pyranoid rings, respectively. The bands near 1640 cm^−1^ were assigned to C=O stretching vibrations of the carboxyl group, indicating the presence of organic or amino acids [36]. The band between 711.60 cm^−1^ and 912. 16 cm^−1^ indicates the presence of β-glycosidic bonds. The band between 1032.23 cm^−1^ and 1111.76 cm^−1^ arose from the C-O stretching vibration of the glycosidic bond.

#### 2.6.3. GC-MS Chromatogram

The GC-MS chromatograms of the compounds present in the mushroom extracts are listed in Table 5.

**Figure 5:**
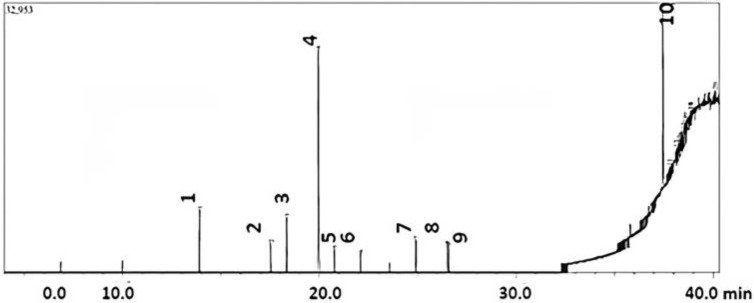
GC-MS chromatogram of *G. applanatum* methanolic extract. Numbers on the peaks indicate individual bioactive compounds listed in Table 5.

Seventeen metabolic compounds were determined from the methanolic extract of *G. applanatum*. Among them, nine compounds were reported in previous studies, whereas eight compounds were newly identified in this study. Among them, 1,2-benzenedicarboxylic acid and diethyl ester were the most abundant compounds (peak area 29.53% and RT of 19.942), followed by 1,4-benzenedicarboxylic acid and bis(2-ethylhexyl) ester (peak area % 23.9 and RT 37.46) (Figure 5).

Some other important compounds, which were much less in abundance, were 2, 3, 4, 4-tretrapropyl-1-(trimethylsilyl)-1-(trimethylsilyloxy)-1,3-diaza-2,4-diborabutane, 2-tert-butyl-4-(1,1,3,3-tetramethylbutyl) phenol, 1,2-benzenedicarboxylic acid, dicyclohexyl ester, 2-cyclobuten-1-one, 4-[[(1,1-dimethylethyl)dimethylsilyl]oxy]-2,3-dimethoxy-4-(3-phenyl-1-propynyl), Furo [2,3-c]pyridine, 2,3-dihydro-2,7-dimethyl, 2H-3,11c-(epoxymethano)phenanthro[10,1-bc]pyran, picras-3-en-21-oic acid deriv, 1-(3,4-ditrimethylsiloxyphenyl)-2-isopropylaminoethanol, 12-azabicyclo(9.2.1)tetradeca-1(14)-ene-13-one, 7-oxabicyclo[2.2.1]hept-2-ene, 5,6-bis(chloromethyl)-2,3-dimethyl-, (exo, exo), isopropenyl dodecanoate, 12-azabicyclo(9.2.1)tetradeca-1(14)-ene-13-one, silane, trimethyl [2-methylene-4,4-bis(phenylsulfonyl)butyl], 2-propanone, 1,1,1-tris(ethylthio)-3-(4-methoxyphenyl)-3-[(trimethylsilyl)oxy], 3-bromo-4-(difluoromethyl)pyridine, cyclotrisiloxane, hexamethyl, 1H-Furo[3,4-c]pyrrole-4-carboxylic acid, 6-(2-furanyl)hexahydro-1,3-dioxo-4-phenyl, methyl ester, (3a.alpha.,4.beta.,6.beta.,6), and 3-pyridinemethanol, and 4-[(5H-dibenzo[a,d] cyclohepten-5-ylimino)methyl]-5-hydroxy-6-methyl-(e)-. The percentage peak area of each metabolite is shown in the GC-MS chromatogram (Figure 5).

### 2.7. Antimicrobial Potential of G. applanatum Mushroom

**Figure 6:**
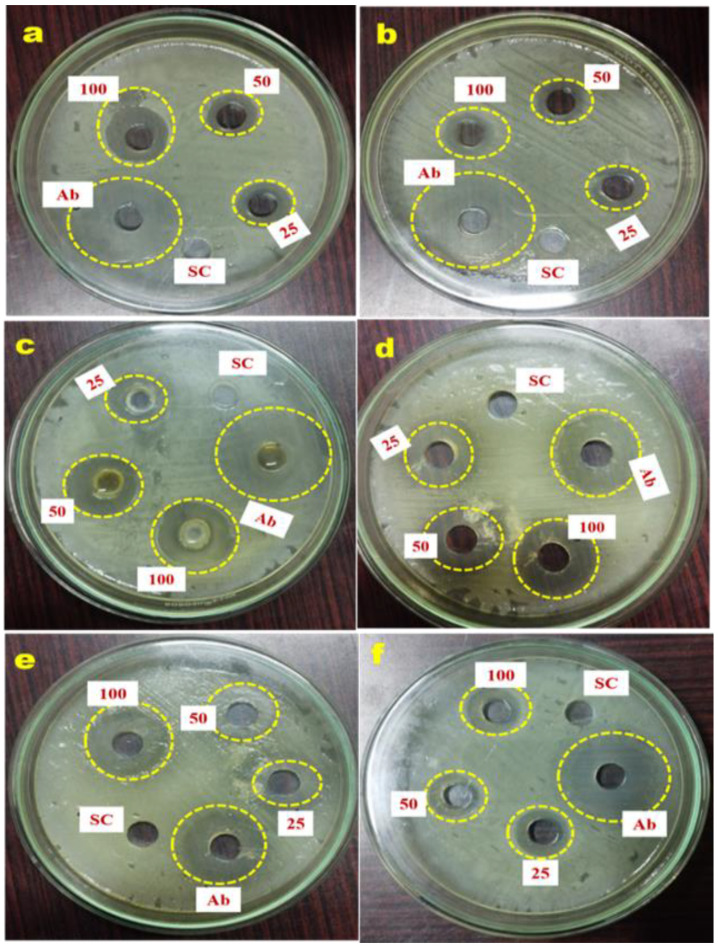
Agar well diffusion technique exhibiting the antimicrobial effects of *G. applanatum* mushroom extract against selected human pathogens: (**a**) *S.* pyogenes, (**b**) *E. faecalis*, (**c**) *K. pneumoniae*, (**d**) *S. flexneri*, (**e**) *C. albicans*, and (**f**) *A. fumigatus*. Various dosages (25, 50, and 100 µg mL^−1^) of *G. applanatum* mushroom extract were analyzed along with positive control (streptomycin for bacteria and griseofulvin for fungi) and solvent control (methanol; 50 μL) against six selected human pathogens. Note: Ab, antibiotics (streptomycin for bacteria and griseofulvin for fungi); SC, solvent control.

In this study, the *G. applanatum* mushroom extract at the dosage of 100 µg mL^−1^ exhibited the highest antimicrobial efficacy against Gram-negative bacterial pathogens by zone of inhibition size (*Shigella flexneri* 19.98 ± 0.88 mm; *Klebsiella pneumoniae* 18.29 ± 1.12 mm) compared with Gram-positive pathogens (*Streptococcus pyogenes* 17.32 ± 0.33 mm; *Enterococcus faecalis* 11.21 ± 0.88 mm) (Figure 6; Table 6).

This is due to structural dissimilarities in the bacterial cell wall, the presence or absence of outer membranes, and the presence or absence of specialized machinery for antibiotic resistance [37,38]. Mushroom extracts showed significantly better antifungal activity against both fungal pathogens (*Candida albicans* 16.54 ± 1.12 mm; *Aspergillus fumigatus* 11.27 ± 0.33 mm) (Table 6). The current study showed that *G. applanatum* mushrooms were capable of inhibiting one or the other of Gram-negative or Gram-positive bacteria, the yeast *C. albicans*, and the mold *A. fumigatus*.

### 2.8. Antioxidant Activity of G. applanatum Mushroom Extract

#### 2.8.1. Radical Scavenging Activity

**Figure 7:**
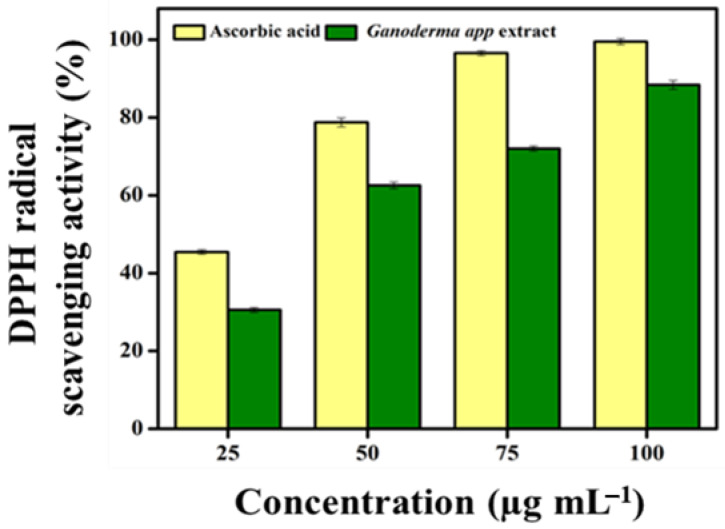
DPPH free radical scavenging activity of *G. applanatum* mushroom extract. Values are expressed as mean ± SD from *n* = 3.

In this study, the methanolic extract of *G. applanatum* scavenged 1,1-diphenyl-2-picrylhydrazyl (DPPH) radicals in a dose-dependent manner (Figure 7). The maximum amount of DPPH radical scavenging was observed at 100 µg mL^−1^ (88.38 ± 1.12%). Based on the IC_50_ value of *G. applanatum* at 50 µg mL^−1^, the DPPH radical scavenging potential was 62.55 ± 0.87%. Compared to a previous report [39], our sample had greater DPPH radical scavenging potential, suggesting that ~500 µg mL^−1^ of mushroom extract inhibits DPPH radicals by up to 40–60% 

#### 2.8.2. Reducing Power Activity

**Figure 8:**
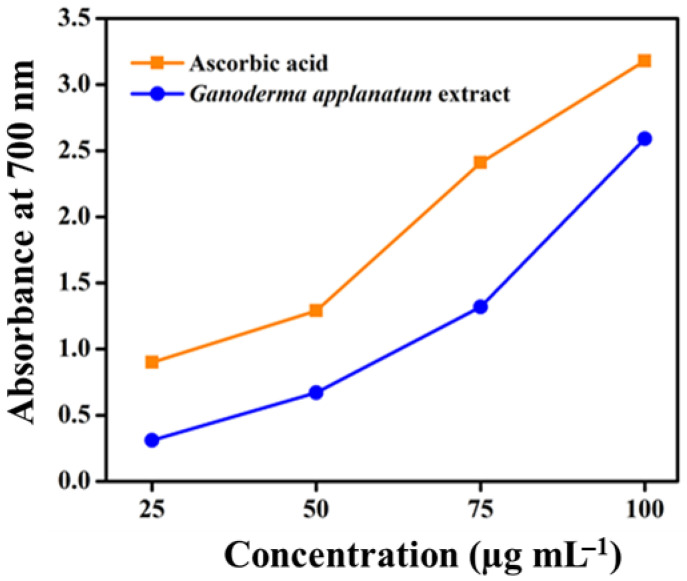
Reducing power efficacy of *G. applanatum* mushroom extract. Values are expressed as mean ± SD from *n* = 3.

The reducing power of the extracts from the wild mushroom *G. applanatum* was determined by measuring the absorbance at 700 nm. The reducing power of the mushroom samples increased in a concentration-dependent manner (Figure 8). By expressing the IC_50_ value, the concentration of the extract showed an absorbance of 0.5 and the highest reducing power was recorded at 100 µg mL^−1^. The results also showed a positive correlation between antioxidant potential and total phenolic content. This may be due to the reaction specificity of the phenolic compounds present in the samples.

## 3. Discussion

Mushrooms are essential for the treatment of various degenerative disorders; therefore, they have long been highly regarded and valued. Moreover, their distinct flavor, taste, and high protein content have increased the demand for nutritious foods. Further details on their therapeutic potential, dietary significance, and chemical constituents will be useful for exploring their value as nutraceuticals. There is a wide variety of physiological efficacies governed by mushrooms. The present study investigated the nutritional profile, elemental analysis, proximate content estimation, and phytochemical analysis of *G. applanatum* mushrooms for their utilization as functional foods and medicines.

A random distribution of samples at the selected sites was considered for sample collection. The opportunistic sampling method involved walking over collection sites and collecting samples from almost 80% of the target area. The climatic and edaphic conditions of the Western Ghats region of Kerala favor the occurrence of diverse mushroom species with seasonal variations. Frequent rainfall, high humidity, optimum temperature, and acidic soil pH are crucial factors that support mycelial development and fruiting body production [40]. The availability of dense forest areas in the entire KFRI region also helps maintain the diversity of wood-rotting fungi (Basidiomycota). Many previous studies support this finding, as different mushrooms grow in different habitats [41,42].

Proximate and qualitative analyses of the extract of *G. applanatum* growing from Kerala were evaluated in this study, and the results were reported by other authors in different countries where different values were observed in the present study; in some cases, these samples had higher values than in other places. However, very few studies have reported the nutritional profile of *G. applanatum*. Malnutrition is a major health issue in emerging nations. Owing to their high per-unit yield, nutritional content, texture, and flavor, mushrooms are an excellent food source for reducing starvation in underdeveloped nations. Carbohydrate and protein contents of 33–38% and 20–30%, respectively, are presented in various types of edible mushrooms, as documented in earlier research [3,4,7]. One of the most vital elements in nourishment is protein, which is crucial for the development of bodily tissues. Owing to their high protein content, mushrooms are a valuable supplement to a person’s diet when animal sources are scarce [43,44]. Insoluble fibers aid in digestion, whereas soluble fibers combat cardiovascular diseases by lowering cholesterol levels. In addition, mushrooms are rich in dietary fiber, which is also known for its anti-tumorigenic and hypocholesterolemic properties. Nagaraj et al. reported moisture content (42% *w*/*w*) results relatively higher than those in the present study [45]. The moisture content should be kept at a minimum to prevent microbial development and unfavorable enzymatic efficacy, which can cause spoiling. The carbohydrate (33.63 ± 0.66%) and crude fiber (16.34 ± 0.33%) contents were quite similar, but the protein (21.01 ± 0.88%) and ash (10.64 ± 0.66%) contents were higher than those reported in previous studies [4,34].

Mushroom extracts contain phenolic substances, which are considered one of the most vital natural forms of antioxidants and are frequently present in mushrooms and plants [46]. Consequently, since phenols and flavonoids have been shown to possess various antioxidant properties, mushrooms can be used to treat diseases caused by oxidative stress. These compounds have been shown to exhibit antimicrobial properties through microbial adhesion, the suppression of protein synthesis, proteolytic enzymes, and the rupture of cell membranes [3,4,47].

Mineral content plays a key role in the development of mushroom fruiting bodies [48]. Singh et al. documented that the elemental analysis of *G. lucidum* shows that the Ca, Na, Zn, and P are significantly similar to our present finding, but the K (23.9 ± 0.66 mg kg^−1^) and Fe (4.83 ± 0.33 mg kg^−1^) are much lower than in *G. lucidum* [40] and the amount of Mn (90.73 ± 1.22 mg kg^−1^) present is higher in *G. applanatum*. This is comparable to other previously studied results that also recommended Mn as the most abundant mineral among samples of different species. Therefore, *G. applanatum* is the best source of Mn and protects the cardiovascular and cerebrovascular systems [14]. As indicated by earlier research, mineral concentrations of mushrooms are significantly influenced by developing media, habitats, and several other internal and external elements, such as growth circumstances and genetic variation [48]. The consumption of mushrooms with high K content maintains the salt balance in the body and regulates blood pressure; it also maintains cardiovascular health. Earlier studies have documented that human uptake reduces the bioavailability of some metals, particularly Cu, owing to inadequate absorption from the small intestine [49]. 

The different proportions of the solvent systems used in column chromatography resulted in fractions obtained from 100% ethyl acetate and hexane/ethyl acetate (5:5 and 8.5:1.5, respectively), which showed the presence of flavonoids. Five different fractions were eluted by column chromatography using hexane/ethyl acetate (5:5 and 8.5:1.5) and 100% ethyl acetate as the solvents. A few earlier TLC reports stated that other linked species of *A. bisporus* and *Pleurotus florida* also contain alkaloids and terpenoids that possess antimicrobial efficacy against both Gram-negative and Gram-positive bacterial strains [2,3]. Therefore, alkaloids and terpenoids may play key roles in suppressing the growth of Gram-negative and Gram-positive pathogenic bacterial strains. To confirm this, the TLC purified fractions of the crude extracts were individually tested against the bacterial strains. Previous reports have stated that *A. bisporus* mushrooms also contain phenolic substances with antioxidant activities [4,36].

A comparison of the R_f_ values of the compounds in the sample with the reference substance revealed several important observations. Notably, the R_f_ values obtained for the tested compounds generally aligned with the R_f_ values of the expected compounds, indicating successful separation and identification. Caffeine is the reference substance for the alkaloid group, exhibiting an R_f_ value of 0.53 [50]. Terpineol is the reference substance for terpenoids with an R_f_ value of 0.56 [51]. This correspondence strengthens the evidence for the presence of flavonoids in the mushroom samples of this study. Furthermore, TLC analysis revealed a compound with an R_f_ value of 0.81, which matches the expected R_f_ value of rutine in this alignment supports the identification of flavonoids in the sample, contributing to our understanding of its chemical composition. Leela and Devi have already documented that glycosides from lichen *Parmelia Perlata* have an R_f_ value of 0.15, which is similar to our result [52]. Similarly, Sonam et al. reported that a compound with an R_f_ value of 0.8 from *Reinwardtia indica* is similar to the current result [53].

FTIR spectroscopy was used to identify the functional groups in the samples. In this study, amide, aldehyde, and carboxylic acid groups were obtained from heterocyclic substances, such as proteins found in *P. florida* mushroom extracts [3]. All of the samples had characteristic peaks that indicated the presence of alkaloids, polysaccharides, proteins, and phenolics. The findings of the present study, including C-O stretching in flavonoids and phenols, were consistent with the quantitative examination of total phenolic and flavonoid compounds. The phenolic and amide-specific peaks found in the FTIR spectrum of the *A. bisporus* mushroom extract strongly correlated with the previously documented antioxidant and antimicrobial potential [4,36].

GC-MS was used to identify the various compounds present in mushrooms. Comparing the mass spectra, retention times, areas, peak heights, and the sample data’s area-to-height ratio of the sample data with the NIST GC-MS library helped identify the substances included in the extract. The compounds identified through GC-MS have different biopotential properties, including anti-inflammatory, antioxidant, anticancer, and antimicrobial effects [54,55]. Most Ganoderma spp. have been reported to contain many biologically active compounds such as phenolics, alkaloids, saponins, flavonoids, fatty acids, and saponins [46,56]. According to Pal et al., compound 1,2 benzene dicarboxylic acid (19.35%) is found in *Astraeus hygrometricus*, which helps in neurotoxicity, anticancer, antiviral, and antibacterial activities [57,58]. Isopropenyl dodecanoate is an odorous compound first reported in *G. applanatum*. Meanwhile, *Ganoderma* sp. contains 1,2-benzenedicarboxylic acid and dicyclohexyl ester, showing potent antibacterial activity [59].

Wild mushrooms possess antibacterial and antifungal properties that allow them to thrive in their native environment. Thus, wild mushrooms have abundant supplies of naturally occurring phytochemical agents. Initial screening for the antimicrobial activity of methanolic *G. applanatum* extracts of wild mushrooms was carried out using the agar well diffusion technique. Extracts containing bioactive compounds diffused out of the well through the porous agar matrix and inhibited the growth of the target bacteria and fungi around the well, as shown in Figure 6. Menaga et al. reported that the control of Gram-negative bacteria, especially cell-wall-suppressing antimicrobial agents [2], is tougher than Gram-positive bacteria. Therefore, the wild mushrooms studied possessed broad-spectrum antibiotics. Although inhibition by bacteria and yeast was not up to the standard antibiotics, these wild mushrooms were capable of inhibiting a broad range of pathogenic microorganisms. Krishnamoorthi et al. showed that the phenolic and tannin contents of *A. bisporus* have antimicrobial properties through microbial adhesion, the suppression of protein synthesis, proteolytic enzymes, and the rupture of cell membranes [4,60].

It has been noted by many researchers conducting different studies that mushrooms are an excellent source of antioxidant compounds. Many researchers have recommended that antioxidant compounds extracted from different mushrooms exhibit protective properties at different phases of the oxidation process via several mechanisms [61]. However, high concentrations, such as 5–20 mg mL^−1^, can inhibit 40–60% of DPPH free radicals in a few edible mushroom species, as reported previously [62]. The solvents used in the extraction process play a vital role in determining the antioxidant properties of biological samples. Previous studies have reported the use of methanol and ethyl acetate as extraction solvents to obtain antioxidant compounds with high antioxidant activity [63]. In this study, *G. applanatum* mushroom extract had a wider number of antioxidant compounds such as flavonoids, phenols, and alkaloids, and scavenged free radicals and the IC_50_ value of *G. applanatum* at 50 µg mL^−1^, especially 2-tert-butyl-4-(1,1,3,3-tetramethylbutyl) phenol compound, had a higher radical scavenging potential. According to Herawati et al. [64], different wild mushrooms such as *Trametes versicolor*, *Microporus xantophus*, *Schizophyllum commune*, and *Auricularia auricula* reported a lower IC_50_ value (494.455, 251.200, 121.046, and 499.249 µg mL^−1^, respectively) than the present study. They also showed that bioactive compounds affect antioxidant capacity. This is strongly associated with the influence of the habitat where they grow [64]. The capacity of a compound to transport electrons is related to its reducing power; therefore, it is considered a significant measure of its antioxidant activity. The presence of a chemical substance with antioxidant capability causes the reduction of the ferric cyanide complex to its ferrous form, resulting in a color change in the reaction mixture from yellow to greenish-blue, based on the ability to reduce the power of each reaction mixture [65]. This is demonstrated by the fact that antioxidant activity increases with increasing environmental stress [66]. A comparison of *G. applanatum* with other wild mushrooms showed greater nutritional value and mildly strong antioxidant activity. Additional research on mushrooms cultivated using diverse substrates must be conducted to assess their medicinal use.

## 4. Materials and Methods

### 4.1. Sample Collection

The fruiting bodies of *G. applanatum* grown on dead and unknown stems were obtained from the Kerala Forest Research Institute, Thirussur, Kerala, India. Samples were collected using a sterile knife, digging apparatus, and pluckers. The collected samples were authenticated by Dr. Shambhu Kumar, Senior Scientist, Department of Forest Pathology, Forest Health Division, Kerala, India. The collected fungal fruiting bodies were kept clean, dry (40–50 °C for 2 days), and powdered, with labeled polythene bags. The collected fungal fruiting bodies were subjected to lyophilization for dehydration and were preserved for long-term storage. Parts of the samples were pulverized using a mixer grinder and stored for further study.

### 4.2. Methanol Extraction from G. applanatum

Methanol extraction from *G. applanatum* mushrooms was performed using a previously reported protocol [67]. Briefly, 30 g of finely ground mushroom powder was extracted with 70% methanol for 8–10 h using Soxhlet extraction apparatus. The extract was then filtered through a Whatman No.1 filter paper. The resulting filtrate was subjected to rotary vacuum evaporation at 40 °C to eliminate the solvent, resulting in a concentrated extract of *G. applanatum*. The concentrated extract was lyophilized using a lyophilizer (Labconco Corporation, Kansas City, MO, USA) and stored at −20 °C.

### 4.3. Proximate Analysis of G. applanatum

#### 4.3.1. Estimation of Carbohydrate

The carbohydrate content of dried *G. applanatum* mushrooms was measured using a standard procedure [68]. Dried mushrooms (0.1 g) with 5 mL of 2.5 N hydrochloric acid (HCl) were placed in a test tube and boiled in a water bath for 3 h. After cooling to room temperature, the reaction mixture was maintained at pH 7 using solid sodium carbonate. This was conducted gradually until fizzing (effervescence). The reaction mixture was spun at 4000 rpm for 5 min and the resulting supernatant (0.5 and 1.0 mL) was used for further carbohydrate analysis. Standard carbohydrate solutions were prepared in a range of concentrations (0–1.0 mL) to create a calibration curve. The volume of each standard and sample tube was adjusted to 1 mL of distilled water (DW), and 4 mL of anthrone reagent was added to each tube, which reacted with carbohydrates to produce a green to dark green color. The tubes were then heated in a boiling water bath for 8 min and quickly cooled, and the intensity of the developed color was measured at 630 nm using a spectrophotometer.

Carbohydrate (%) = [Mg of glucose/volume of the sample]/100

#### 4.3.2. Estimation of Protein

DW (10 mL) was used to dissolve 5 mg of bovine serum albumin (BSA), and ammonium sulfate was gradually added, equaling 50–100% of its saturation level to the BSA solution, ensuring that the mixture was stirred continuously. Once ammonium sulfate was fully incorporated, the mixture was left to stand undisturbed for 30 min. The sample was then spun at 11,000 rpm for 5 min. The supernatant was discarded, and the pellet was resuspended in DW. This reaction was repeated twice and the pellet was resuspended in 1 mL of DW. From this final suspension, 200 µL of the sample was used for protein quantification using the Lowry method.

Recovery (%) = (Ending protein content/Starting protein content) × 100

#### 4.3.3. Estimation of Moisture

The moisture concentration of powdered *G. applanatum* mushrooms was assessed following the standard procedure outlined in the AOAC [68]. Initially, 10 g of sample was dried at 105 °C for 6 h. The process continued until the sample reached a consistent weight, indicating the complete removal of moisture. The moisture contents of the samples were determined using the following formula:

Moisture level (%) = [(Starting weight − Ending weight)/weight of sample] × 100

#### 4.3.4. Estimation of Fat

The fat content of dried *G. applanatum* mushrooms was assessed following the standard procedure outlined by the AOCC [68]. Dried mushroom samples (5 g) were placed inside a thimble in an extraction tube in a Soxhlet apparatus. The heater temperature was adjusted to ensure continuous drip of ether on the sample within the extraction tube. The extraction process used petroleum ether with a boiling point range of 40–60 °C and lasted for 16 h. After completion of the extraction, the sample was removed and the solvent was allowed to evaporate under a fume hood. The resulting extract was thoroughly dried in a hot air oven for 30 min at 105 °C. After cooling in a desiccator, the weight of the extracts was recorded. The determination of crude fat content was subsequently carried out using the following formula:

Crude Fat (%) = [Weight of sample/Weight of Fat in sample] × 100

#### 4.3.5. Estimation of Ash Content

The procedure for estimating the ash content of *G. applanatum* mushrooms was performed in compliance with the methodology specified by the AOAC [68]. Initially, the crucible with its lid was heated in a furnace at 550 °C overnight and then cooled in a desiccator for 30 min. The cooled crucible, still with its lid, was precisely weighed to three decimals. Approximately 5 g of mushrooms was added to the crucible. The sample was then gently heated using a Bunsen burner with a lid partially covering the crucible, until no additional smoke was produced. The crucible, which was fully covered with a lid, was placed back in the furnace and heated overnight at 550 °C. After heating, the crucible was covered with a lid and cooled in a desiccator. This process helps to remove all organic material through combustion, leaving behind inorganic ash for measurement. The ash level was determined using the following formula:

Ash content (%) = [Weight of sample/Weight of ash sample] × 100

#### 4.3.6. Estimation of Crude Fiber

Crude fiber content was assessed using a method involving the sequential extraction of *G. applanatum* as outlined in the AOAC [68]. The extraction process involved the use of 1.25% sulfuric acid (H_2_SO_4_) and 1.25% sodium hydroxide (NaOH) solutions, utilizing a fiber bag as a container for the samples. Following the extraction, the samples were dried and ashed for further analysis. The samples were dried in a crucible for 5 h at 105 °C, and subsequently ashed in a muffle furnace overnight at 525 °C.

Crude Fiber (%) = [Weight of sample/loss of weight after ignition] × 100

#### 4.3.7. Estimation of Energy Values

The energy values were investigated using the following equation:

Energy values (Cal/100 g) = (4.2 × % carbohydrate) + (8.37 × % fat) + (2.62 × % proteins)

### 4.4. Preliminary Phytochemical Analysis Using Biochemical Methods

*G. applanatum* mushroom extract was subjected to phytochemical screening using various known qualitative biochemical tests, as used in a previous report, with minor modifications [69].

#### 4.4.1. Phenol Test (Lead Acetate Test)

A few drops of a 1% lead acetate solution was mixed with 1 mL of the methanolic mushroom extract. The production of a bluish-black cloud showed the existence of phenolic compounds.

#### 4.4.2. Flavonoid Test (Alkaline Reagent Test)

A few drops of NaOH solution were added to the mushroom extract. The emergence of an intense yellow color, transitioning to colorlessness upon adding a few drops of diluted acid, proved that flavonoids were present.

#### 4.4.3. Terpenoid Test (Salkowski Test)

The mushroom extract underwent treatment with a few drops of concentrated H_2_SO_4_, followed by thorough shaking and standing for a specific duration. The appearance of a yellow-colored lower layer was indicative of the presence of triterpenoids.

#### 4.4.4. Steroid Test

A few drops of strong H_2_SO_4_ were added to the mushroom extract, which was then thoroughly mixed and let to stand for a while. The emergence of red color in the bottom layer indicated the existence of steroids.

#### 4.4.5. Alkaloid Test (Mayer’s Test)

A few drops of Mayer’s reagent were added to 5 mL of mushroom extract. The reactive solution exhibited a indicated a white, creamy precipitate, indicating a positive result.

#### 4.4.6. Test for Tannins (Ferric Chloride Test)

A few drops of ferric chloride were mixed to 1 mL of the mushroom extract. The emergence of a deep bluish-black color suggested the existence of tannins.

#### 4.4.7. Saponin Test (Frothing Test)

Here, 1 mL of mushroom extract was added to 4 mL of distilled water. The mixture was shaken and maintained for at least 15 min, and the presence of continuous foam indicated the presence of saponin.

### 4.5. Quantitative Estimation of Phytochemicals from G. applanatum Mushroom Extracts

#### 4.5.1. Total Amount of Flavonoid

The colorimetric technique using aluminum chloride was used to determine the total amount of flavonoid in the *G. applanatum* mushroom extract [70]. Briefly, 0.5 mL of mushroom extract was mixed with 2.8 mL of DW, 0.1 mL of 1 M potassium acetate, 0.1 mL of 10% AlCl_3_, and 1.5 mL of methanol for 30 min at room temperature. After incubation, the intensity of the resulting mixture was calculated using the absorbance values at 415 nm. Quercetin was used as a standard. The extracts’ total flavonoid content (TFC) was determined from the quercetin calibration graph using the formula T = C × (V/M). Here, T stands for TFC (mg g^−1^), C is the quercetin concentration (mg mL^−1^) obtained from the calibration graph, V is the extract volume (mL), and M is the extract weight (g). The milligrams of quercetin equivalent (QE) per gram of sample dry weight indicates how the TFC is stated.

#### 4.5.2. Total Amount of Terpenoid

The total terpenoid content of phenolics in the *G. applanatum* mushroom extract was assessed using the standard technique, as previously described [71]. Briefly, 100 mg of mushroom extract was immersed in 9 mL of ethanol for 24 h and then filtered through a Whatman No.1 filter paper. The resulting solution was subsequently extracted using a separating funnel containing 10 mL petroleum ether. The resulting ether extract was then divided into pre-weighed glass vials and allowed to undergo thorough drying until completion. The dried ether extract was considered the total terpenoid content.

#### 4.5.3. Total Amount of Phenolics

The total amount of phenolics in the *G. applanatum* mushroom extract was determined using Folin–Ciocalteu reagent [72]. Briefly, Folin–Ciocalteu reagent (2.5 mL) was thoroughly mixed with a 0.5 mL aliquot of the extract. After a 5 min interval, 2 mL of 7.5% sodium carbonate (Na_2_CO_3_) solution was added to the reaction mixture and incubated at 45 °C for 15 min. The reaction mixture was developed in blue and the absorbance was measured at 765 nm. Gallic acid served as a standard, and the total phenolic content (TPC) was determined from the validation plot of gallic acid. The TPC of the extract was calculated using the formula T = C × (V/M), where T represents the TPC (mg g^−1^) of the mushroom extract, C shows the dose of gallic acid (mg mL^−1^) obtained from the validation plot, V represents the volume of the mushroom extract obtained (mL), and M is the weight of the mushroom extract (g). The results are represented as mg of gallic acid equivalents (GAE) per gram of sample dry weight.

#### 4.5.4. Total Saponin Content

To determine the total saponin content of *G. applanatum* mushrooms, a vanillin–sulfuric acid assay was performed using a standard procedure. Briefly, 2.5 mL of 72% (*v*/*v*) H_2_SO_4_ in water, reagent blank or standards with 0.25 mL of 0.8% (*w*/*v*) vanillin in ethanol and 1 mg ml^−1^ of mushroom extracts were kept in a shaking water bath at 60 °C for 15 min. Diosgenin was used as the standard, and methanol as the reagent blank. The content of the mushroom extracts and standard was determined by the absorbance at 544 nm using UV-Vis spectroscopy [71].

### 4.6. Elemental Analysis

One gram of methanolic *G. applanatum* mushroom extract was subjected to ash in an oven at 650 °C for a duration—5–6 h. Subsequently, it was dissolved in HNO_3_, followed by digestion with H_2_O_2_ and HCl. The solution was allowed to cool and then centrifuged. After centrifugation, the solution was diluted to 10 mL using distilled H_2_O_2_. Elemental analysis was performed using an Atomic Absorption Spectrophotometer (AAS) [73].

### 4.7. Compound Elucidation and Characterization

#### 4.7.1. Thin-Layer Chromatography (TLC)

TLC was performed to identify the different compounds present in the *G. applanatum* mushroom extract as per the standard procedure [74]. A suitable developing solvent (mobile phase) was prepared based on the compounds used for analysis. The mobile phase was prepared by dissolving different solvents for different bioactive compounds, such as alkaloids [chloroform/methanol/toluene (4:2:2)], flavonoids [chloroform/methanol/toluene (33:7:10)], terpenoids [toluene/chloroform/ethanol (40:40:10)], glycosides [toluene/chloroform (9:1)] and phenols [petroleum ether/ethyl acetate (7:3)]. After the chromatogram was developed, the retention factor (Rf) was calculated using the formula. The target compounds were purified using column chromatography.

#### 4.7.2. Column Chromatography

This technique was performed using silica gel (Nice—60–120 Mesh), as per the protocol [74]. The methanol extract was then chromated using a silica gel column. Initially, the column was eluted with hexane (100%), followed by hexane/ethyl acetate (10:1), hexane/ethyl acetate (1:5), hexane/ethyl acetate (1:10), ethyl acetate (100%), ethyl acetate/methanol (10:5), ethyl acetate/methanol (1:5), and methanol (100%).

#### 4.7.3. Fourier-Transform Infrared Spectroscopy (FTIR)

FTIR spectra were employed for the characterization of *G. applanatum* mushroom using a methanol extract sample through Attenuated Total Reflectance (ATR) in the area of 400–4000 cm^−1^. Functional groups were identified using an Alpha-II FTIR-ATR instrument provided by Bruker International [4].

#### 4.7.4. Gas Chromatography–Mass Spectrometry (GC-MS)

The secondary bioactive compounds presented in the methanol extract of *G. applanatum* were screened using GC-MS and recorded on a SHIMADZU/QP2020 equipped with a column model of SH-RXi-5Sil MS as per the standard procedure [67]. Helium was used as the flow gas. The column temperature mode was 50 °C at the initial point, and then changed to 280 °C at a rate of 6.00 °C for a holding time of 2 min. By comparing the retention times, areas of proportion, and mass spectra fragmentation patterns of the chemicals in the mushroom extracts to those kept in computer libraries and in the published literature, the identities of the constituents were determined. The chemicals that were determined were matched using NIST08s.LIB and WILEY8.LIB library sources.

### 4.8. Antimicrobial Activity of Methanolic G. applanatum Mushroom Extract

The antimicrobial activity of the methanolic crude *G. applanatum* mushroom extracts was determined against six selected human pathogenic strains [two fungal pathogens: *A. fumigatus* (MTCC 2550) and *C. albicans* (MTCC 3017), and four bacterial pathogens: *E. faecalis* (MTCC 439), *S. flexneri* (MTCC 1457), *K. pneumoniae* (MTCC 432), and *S. pyogenes* (MTCC 442)] using the well diffusion method [60]. The pathogen was procured from the Microbial Type Culture Collection (MTCC; Chandigarh, India). The bacterial and fungal strains were pre-grown in Nutrient Broth (NB) and potato dextrose broth (PDB). To assess antimicrobial activity, Mueller–Hinton agar (MHA) plates were prepared and spread on pathogenic strains on the surface and the wells were made (6 mm diameter) using a sterile well borer. Following this, the different dosages (100, 50, and 25 μg mL^−1^) of the methanolic mushroom extracts were loaded into the respective wells of the plates along with 50 μg mL^−1^ of positive control (streptomycin for bacteria and griseofulvin for fungi) and negative control methanol (50 μL) against each tested pathogenic strain, and the plates were incubated for 24 h at 37 °C. A zone of inhibition encircling the wells suggested that positive antibacterial activity was observed. Three replicates were carried out for this examination.

### 4.9. Antioxidant Activity of Methanolic G. applanatum Mushroom Extract

#### 4.9.1. Free Radical Scavenging Assay

The methanolic *G. applanatum* mushroom extract was used to assess DPPH free radical scavenging potential using previously described methods [4]. Different dosages (25, 50, 75, and 100 μg mL^−1^) of the methanolic mushroom extract combined with 1 mL of DPPH solution were kept at 28 °C for 30 min in dark. Methanol served as the blank, the control consisted of DPPH in methanol without the sample, ascorbic acid was utilized as the standard reference, and absorbance at 517 nm (Ab_517_) was recorded. The DPPH radical scavenging capacity was determined using the following formula:

% DPPH scavenging potential = [(Ab517 (control) – Ab_517_ (sample)/Ab_517_ (control)) × 100, where Ab_517_ (control) and Ab_517_ (sample) is the absorbance of DPPH.

#### 4.9.2. Reducing Power Assay

The reduction power was assessed in accordance with the previous method [65]. Various dosages (25, 50, 75, and 100 μg mL^−1^) of the methanolic *G. applanatum* mushroom extract were combined with 1.5 mL of 1% potassium ferricyanide and 1.5 mL of 0.2 M sodium phosphate buffer (pH 6.6). The reaction mixture was mixed with 5 mL of 10% trichloroacetic acid and incubated for 20 min at 50 °C. After 20 min, the reaction mixture was centrifuged at 8000 rpm at 4 °C for 7 min. Then, 1.5 mL of the supernatant was taken from the top layer, 0.1% ferric chloride (300 μL) and DW (1.5 mL) were added, and the absorbance was measured at 700 nm. Ascorbic acid was used as a reference material. An increase in the absorbance within the resultant mixture signified a higher level of reducing power. The concentration at 0.5 absorbance was determined to be the benchmark for evaluating the scavenging capacity.

### 4.10. Statistical Analysis

Statistical computations were performed using Origin 8.1 (Origin Lab Corporation, Northampton, USA), and the mean of three replicates ± standard deviation was displayed as the outcome. The Student’s *t*-test for dataset was used to determine the *p*-value, and *p* < 0.05 was regarded as statistically significant.

## 5. Conclusions

In conclusion, this study provides a comprehensive analysis of *G. applanatum* By focusing on proximate composition, elemental content, chemical composition, and bioactivity. Proximate analysis revealed that *G. applanatum* is rich in carbohydrate (33.63 ± 0.66%) and protein (21.01 ± 0.88%), making it a nutritious source. The presence of K (283 ± 3.12 mg kg^−1^), Na (190 ± 2.76 mg kg^−1^), P (117 ± 1.12 mg kg^−1^), and Mn (90 ± 1.22 mg kg^−1^) was confirmed in *G. applanatum* through AAS analysis. Qualitative and quantitative analyses of the bioactive components were performed. This study reported 20 compounds using GC-MS analysis, and of these, 8 unidentified compounds were identified. This presents a great opportunity for further research in this field. The methanolic extract of *G. applanatum* has significant antimicrobial potential against human pathogens such as *S. flexneri* (19.98 ± 0.88 mm), *K. pneumoniae* (18.29 ± 1.12 mm), *S. pyogenes* (17.32 ± 0.33 mm), *C. albicans* (16.54 ± 1.12 mm), *A. fumigatus* (11.27 ± 0.33 mm), and *E. faecalis* (11.21 ± 0.88 mm). *G. applanatum* has significant enrichment with 2-tert-butyl-4-(1,1,3,3-tetramethylbutyl) phenol and may act as a potential therapeutic agent by protecting the biological system from the generation of free radicals, thereby reducing the illness triggered by oxidative stress. The identified compounds, along with their diverse bioactivities, warrant further studies to elucidate their mechanisms and potential health benefits prior to clinical trials.

## Figures and Tables

**Table 1:** Primary phytochemicals of *G. applanatum* using biochemical tests.

Qualitative Analysis of Phyto Compounds			
Sample No.	Biochemical Test	Compound	*G. applanatum*
1	Salkowski test	Triterpenoids	+
2	FeCl_3_ test	Tannins	−
3	Mayer’s test	Alkaloids	+
4	Alkaline reagent	Flavonoids	+
5	Lead Acetate	Phenol	+
6	Frothing test	Saponins	+
7	Keller–Kiliani test	Glycosides	+

Note: (+) indicates presence; (−) indicates absence.

**Table 2:** Quantitative analysis of phytochemicals from *G. applanatum* mushroom.

Sample No.	Biochemical Test	Amount of Phytochemicals per Dry Weight of Mushroom
1	Phenols	22.9 mg GAE per gram
2	Flavonoid	15.84 mg QE per gram
3	Saponin	22.19 µg per milligram
4	Terpenoid	0.351 mg per gram

Note: GAE, gallic acid equivalents; QE, quercetin equivalents.

**Table 3:** Contents of minerals in *G. applanatum* mushroom.

Sample No.	Minerals	Mineral Contents (mg kg^−1^)
1	Copper (Cu)	0.781 ± 0.88
2	Calcium (Ca)	283 ± 3.12
3	Zinc (Zn)	10.34 ± 1.27
4	Potassium (K)	23.9 ± 0.66
5	Manganese (Mn)	90.73 ± 1.22
6	Magnesium (Mg)	4.6 ± 0.66
7	Iron (Fe)	4.83 ± 0.33
8	Sodium (Na)	190 ± 2.76
9	Phosphorous (P)	117 ± 1.12

Note: The amount of each mineral is expressed as mg per kg of mushroom. Values are the mean ± SD from *n* = 3.

**Table 4:** TLC profile of different bioactive compounds in *G. applanatum* mushroom extract.

Lane	Distance Traveled by the Solute (cm)	Distance Traveled by the Solvent (cm)	Band Colour Observed under UV Light	Rf Value	Expected Compound
GA1	2.3	4.3	Green	0.534	Alkaloid
GA2	3.4	4.2	Blue	0.809	Flavonoid
GA3	2.3	4.1	Blue	0.560	Terpenoid
GA4	0.7	4.5	Blue	0.155	Glycoside
GA5	3.1	4.3	Blue	0.720	Phenolic

**Table 5:** GC-MS profile of different bioactive compounds in *G. applanatum* mushroom extract.

Peak	Retention Time (RT)	Peak Area (%)	Name	Molecular Weight (g mol^−1^)	Molecular Formula
1	13.91	5.53	2,3,4,4-tretrapropyl-1-(trimethylsilyl)-1-(trimethylsilyloxy)-1,3-diaza-2,4-diborabutane	UN	UN
2	17.527	2.5	2-cyclobuten-1-one, 4-[[(1,1-dimethylethyl)dimethylsilyl]oxy]-2,3-dimethoxy-4-(3-phenyl-1-propynyl)-	UN	UN
3	18.34	5.84	2-tert-butyl-4-(1,1,3,3-tetramethylbutyl)phenol	262.4	C_18_H_30_O
4	19.942	29.53	1,2-benzenedicarboxylic acid, diethyl ester	222.24	C_12_H_14_O_4_
5	20.766	1.57	1-(3,4-ditrimethylsiloxyphenyl)-2-isopropylaminoethanol	UN	UN
6	22.083	1.04	Isopropenyl dodecanoate	334	C_22_H_38_O_2_
7	24.904	3.04	1,2-benzenedicarboxylic acid, dicyclohexyl ester	330.4	C_20_H_26_O_4_
8	26.493	3.26	Furo[2,3-c]pyridine, 2,3-dihydro-2,7-dimethyl-	149.19	C_9_H_11_NO
9	26.59	0.97	2H-3,11c-(epoxymethano)phenanthro[10,1-bc]pyran, picras-3-en-21-oic acid derivative	548.6	C_28_H_36_O_11_
10	37.46	23.99	1,4-benzenedicarboxylic acid, bis(2-ethylhexyl)ester	390.6	C_24_H_38_O_4_
11	37.783	0.94	3-pyridinemethanol, 4-[(5H-dibenzo[a,d]cyclohepten-5-ylimino)methyl]-5-hydroxy-6-methyl-,(e)-	UN	UN
12	38.145	0.86	Silane, trimethyl[2-methylene-4,4-bis(phenylsulfonyl)butyl]	UN	UN
13	38.273	3.95	2-propanone, 1,1,1-tris(ethylthio)-3-(4-methoxyphenyl)-3-[(trimethylsilyl)oxy]-	UN	UN
14	38.325	5.13	3-bromo-4-(difluoromethyl)pyridine	208	C_6_H_4_BrF_2_N
15	38.48	2.2	7-oxabicyclo [2.2.1]hept-2-ene, 5,6-bis(chloromethyl)-2,3-dimethyl-, (exo,exo)-	UN	UN
16	38.615	4.99	12-azabicyclo(9.2.1)tetradeca-1(14)-ene-13-one	UN	UN
17	38.648	1.46	12-azabicyclo(9.2.1)tetradeca-1(14)-ene-13-one	UN	UN
18	38.86	1.11	12-azabicyclo(9.2.1)tetradeca-1(14)-ene-13-one	UN	UN
19	38.9	1.63	Cyclotrisiloxane, hexamethyl-	222.46	C_6_H_18_O_3_Si_3_
20	40.116	0.48	1H-Furo[3,4-c]pyrrole-4-carboxylic acid, 6-(2-furanyl)hexahydro-1,3-dioxo-4-phenyl-, methyl ester, (3a.alpha.,4.beta.,6.beta.,)	UN	UN

Note: UN, unknown.

**Table 6:** Antimicrobial potential of *G. applanatum* mushroom extract showing the zone of inhibition (well size 6 mm) against six selected human pathogens. Values are mean ± SD from *n* = 3. NA indicates no activity.

Pathogens	Zone of Inhibition (mm) (Well Size 6 mm)				
	Mushroom Extract (µg mL^−1^)			Antibiotic Control	Solvent Control
	25	50	100	(30 µg mL^−1^)	50 µL
Gram-positive bacterial pathogens					
*S. pyogenes*	10.22 ± 0.33	11.33 ± 0.88	17.32 ± 0.33	26.12 ± 0.33	NA
*E. faecalis*	9.98 ± 0.88	10.64 ± 0.66	11.21 ± 0.88	25.9 ± 0.12	NA
Gram-negative bacterial pathogens					
*K. pneumoniae*	9.78 ± 0.88	13.58 ± 0.33	18.29 ± 1.12	26.4 ± 0.33	NA
*S. flexneri*	11.56 ± 0.33	14.27 ± 0.66	19.98 ± 0.88	21.28 ± 0.33	NA
Fungal pathogens					
*C. albicans*	9.1 ± 0.33	12.75 ± 0.88	16.54 ± 1.12	18.21 ± 0.66	NA
*A. fumigatus*	8.97 ± 0.33	9.58 ± 0.66	11.27 ± 0.33	23.9 ± 0.88	NA

## Data Availability

Data available on request.
